# Some Scientific Aspects of Insanity

**Published:** 1892-03-12

**Authors:** 


### Some Scientific Aspects of Insanity.

### XVI.—The Insanities of the Various Periods of Life.

At the crises of the various periods of the great reproductive function fundamental changes occur in the constitution and disposition of the sanest human beings. It may therefore easily be conceived that in those individuals who have the misfortune to inherit an instability of the nervous system the crises of the reproductive functions are liable to be attended with a revolutionary disturbance of the ordinary physiological action of the nervous system. The periods at which disturbances do most frequently manifest themselves with most disastrous results to the individual are (1) puberty and adolescence; (2) pregnancy and the puerperal state; (3) the climacteric period; (4) old age. The subject of senility so far as senile dementia is concerned has been already referred to, and it is unnecessary further to dilate upon it. I shall, therefore, begin with (1) The insanity of adolescence.

In order to understand fully the diseased mental condition which accompany this period of life, it is profitable briefly to observe the ordinary physiological changes to which the nervous system and the mind are subject. The sudden transition which takes place at the age of from 15 to 18, from boyhood and girlhood on the one hand, to manhood and womanhood on the other, is attended by the influx of a multitude of ideas, different methods of observing nature and existence, and the shadowy formation of plans and resolutions for the future of life, which of necessity are unsettling and over-stimulating to the untried mind. Above all, the more correct and expansive ideas of sex are apt to lend a waywardness, self-consciousness, and a certain amount of erratic conduct to the behaviour of the individual—a characteristic common to the lower animals at their breeding seasons.

The period of adolescence is characterised chiefly by an oscillating emotionalism, which is beautifully pourtrayed in the case of the male in Thackeray's "History of Pendennis," and in that of the female by George Eliot in Gwendoline in "Daniel Deronda." The pathological mental manifestations of adolescence are innumerable, and they are apt to vary extremely in the same individual. Indeed, the multiform manifestations of mental aberrations at this period at different stages of its course is one of the characteristics of adolescent insanity. The patient may hover on the borderland, and may eventually escape technical insanity after having been the source of endless trial and grief to his relatives or guardians. On the other hand the affection may declare itself with a very acute attack of mania or melancholia—as a rule when insanity definitely sets in the attacks are apt to be acute. From the attack of mania the patient may recover and lead a listless, aimless, ineffectual existence, when after an interval of from six to eighteen months an attack of acute melancholia passing on to stupor may extinguish his intellectual life.

It may be said generally that insanity occurring at the adolescent period is a very curable disorder. Certainly, the majority of patients recover from the individual attacks. A large percentage of the recoveries would, however, if traced out, be found to have relapses, and a considerable proportion of those afflicted with insanity at this period would be found ultimately to end in secondary dementia. When it is remembered that every adolescent who becomes the victim of the characteristic insanity of that period of life is a person born with an unstable nervous system—in fact the hereditarily degenerate nervous cases might be classified as idiots, imbeciles, and adolescent insane—when this is remembered it will not appear wonderful that an acute attack of insanity should so far affect the unstable, undeveloped, adolescent brain as to render it functionless so far as its most complex workings are concerned.

